# Behavioral health interventions in malaria control: Efficacy and implementation

**DOI:** 10.1097/MD.0000000000043762

**Published:** 2025-08-01

**Authors:** Emmanuel Ifeanyi Obeagu, Getrude Uzoma Obeagu, Michael Unata Iduh

**Affiliations:** a Department of Biomedical and Laboratory Science, Africa University, Mutare, Zimbabwe; b School of Nursing Science, Kampala International University, Ishaka, Uganda; c Department of Medical Microbiology, School of Medical Laboratory Science, College of Health Sciences, UsmanuDanfodiyo University, Sokoto, Nigeria.

**Keywords:** behavioral change communication, behavioral health interventions, community education, community-based interventions, efficacy, implementation, malaria control

## Abstract

Malaria remains a critical global health issue, particularly in regions such as sub-Saharan Africa and Southeast Asia. While advancements in medical technology and pharmaceuticals have made significant contributions to malaria control, the integration of behavioral health interventions is increasingly recognized as essential for a comprehensive approach. This review explores the efficacy and implementation of behavioral health interventions in malaria control, focusing on community education, behavioral change communication, and community-based strategies. Community education initiatives have demonstrated significant success in enhancing awareness and promoting preventive measures such as the use of insecticide-treated nets and early treatment-seeking behavior. Behavioral change communication strategies leverage mass media, interpersonal communication, and social mobilization to foster positive health behaviors and mitigate harmful practices. Community-based interventions, involving local populations in planning and execution, have proven effective in fostering ownership and sustainability, leading to notable reductions in malaria prevalence. Despite their potential, the implementation of behavioral health interventions faces several challenges, including cultural barriers, resource limitations, and inadequate monitoring and evaluation frameworks. Overcoming these obstacles requires tailored approaches that respect local cultural contexts, sustainable funding, and capacity building. Additionally, fostering community involvement, multi-sectoral collaboration, and leveraging innovative communication technologies can enhance the effectiveness and reach of these interventions. By integrating behavioral health strategies with existing medical and technological measures, malaria control programs can achieve more significant and sustainable impacts.

## 1. Introduction

Malaria remains one of the most pressing global health challenges, particularly affecting regions in sub-Saharan Africa, Southeast Asia, and parts of South America.^[1]^ The disease, caused by Plasmodium parasites and transmitted by Anopheles mosquitoes, continues to cause significant morbidity and mortality despite decades of control efforts.^[2]^ In 2022, there were an estimated 229 million malaria cases and 409,000 deaths worldwide, with children under 5 and pregnant women being particularly vulnerable.^[3]^ While technological advancements and pharmacological interventions have made significant strides in malaria control, it is increasingly clear that these alone are not sufficient. A comprehensive approach that includes behavioral health interventions is essential to achieving sustained reductions in malaria transmission and burden. Behavioral health interventions play a crucial role in malaria control by addressing the human behaviors and social determinants that influence malaria transmission and prevention.^[4]^ These interventions include community education, behavioral change communication (BCC), and community-based strategies.^[5]^ By improving knowledge, attitudes, and practices related to malaria prevention and treatment, behavioral health interventions can enhance the effectiveness of other control measures such as insecticide-treated nets (ITNs), indoor residual spraying (IRS), and antimalarial medications. Malaria continues to be one of the world’s most devastating infectious diseases, with the World Health Organization (WHO) estimating 249 million cases and 608,000 deaths globally in 2022. Sub-Saharan Africa bears approximately 94% of the global malaria burden, particularly among children under 5 and pregnant women. Despite these staggering figures, significant progress has been made toward malaria control and elimination, largely due to a combination of biomedical and behavioral interventions.

Community education initiatives aim to raise awareness about malaria transmission, prevention, and treatment among at-risk populations.^[6]^ These programs are typically delivered through schools, community centers, health clinics, and various media platforms, including pamphlets, posters, and digital tools. Effective community education can lead to increased use of preventive measures, timely treatment-seeking behavior, and overall better management of malaria at the community level. BCC strategies are designed to promote positive health behaviors and reduce risky practices that contribute to malaria transmission.^[7]^ BCC employs a range of methods, including mass media campaigns, interpersonal communication, and social mobilization efforts. These strategies are often tailored to specific cultural and social contexts to maximize their impact. For instance, radio and television broadcasts, community theater, and peer education programs have been used successfully in various settings to encourage the use of ITNs, IRS, and prompt treatment. Community-based interventions involve the active participation of local communities in the planning, implementation, and evaluation of malaria control activities.^[8]^ These interventions leverage local knowledge and resources, fostering a sense of ownership and sustainability. Community health workers (CHWs), for example, play a pivotal role in delivering education, distributing ITNs, and administering antimalarial treatments.^[9]^ By engaging communities, these interventions can address local barriers to malaria control and ensure that strategies are culturally appropriate and accepted.

Despite their potential, the implementation of behavioral health interventions in malaria control faces several challenges.^[10]^ Cultural beliefs and practices can influence the acceptance and effectiveness of these interventions. Misconceptions about malaria transmission and treatment can hinder the adoption of preventive measures and timely treatment-seeking behavior. Additionally, resource limitations, including funding, trained personnel, and infrastructure, pose significant obstacles to the implementation and sustainability of these programs. Effective monitoring and evaluation (M&E) systems are essential to assess the impact of behavioral health interventions and guide program adjustments.^[11]^ However, inadequate M&E frameworks can limit the ability to measure outcomes and improve intervention strategies. Ensuring robust M&E systems requires investment in data collection, analysis, and reporting, as well as capacity building for health workers and program managers.

Community involvement is a critical factor in the success of behavioral health interventions. Engaging communities in the design and implementation of interventions enhances their relevance and acceptance.^[12]^ Community involvement fosters trust and ownership, leading to more sustainable and effective malaria control efforts. It also helps to identify and address local barriers to malaria prevention and treatment, ensuring that interventions are tailored to the specific needs and contexts of the communities they serve. Multi-sectoral collaboration is another important facilitator of effective behavioral health intervention. Collaboration between health sectors, educational institutions, nongovernmental organizations (NGOs), and government agencies can enhance the reach and impact of these interventions. Integrated approaches ensure that resources are efficiently utilized and efforts are synergistic. For example, school-based education programs can be supported by health services to provide comprehensive malaria education and prevention. Innovative communication strategies, such as mobile health (mHealth) applications and social media, offer new opportunities to improve the dissemination of health information and engagement with at-risk populations. These technologies provide scalable solutions for reaching wide audiences and facilitating real-time communication and feedback. By leveraging these tools, malaria control programs can enhance the accessibility and effectiveness of their behavioral health interventions.

## 2. Aim

The aim of this review article is to critically evaluate and synthesize existing research on behavioral health interventions in malaria control, focusing on their efficacy and implementation. This review will examine various behavioral health strategies, their impact on reducing malaria incidence, and the challenges and successes associated with their implementation. By doing so, the article intends to identify best practices and provide recommendations for future interventions and research in the field.

## 3. Review methods

This narrative review employed a qualitative synthesis of peer-reviewed literature, official reports, and gray literature to evaluate the efficacy and implementation of behavioral health interventions in malaria control. The review was guided by a problem-focused approach, aiming to bridge the gap between biomedical interventions and behavioral health strategies in malaria-endemic regions. A comprehensive literature search was conducted using databases including PubMed, Google Scholar, and Scopus, covering publications from 2000 to 2024. Key search terms included combinations of “behavioral health,” “malaria prevention,” “community-based intervention,” “ITN use,” “IRS compliance,” “health education,” and “treatment-seeking behavior.” Reports from international health bodies such as the WHO, President’s Malaria Initiative (PMI), and national malaria control programs were also included to ensure policy relevance. Studies and reports were selected based on their relevance to behavioral change strategies and their documented outcomes on malaria control. Both quantitative and qualitative studies were considered, especially those reporting changes in knowledge, attitude, practice (KAP), and health outcomes. Articles focused solely on clinical or pharmacological aspects of malaria without behavioral components were excluded. Thematic analysis was employed to categorize interventions into major domains, such as health education, ITN usage, IRS acceptance, and treatment-seeking behavior. The implementation approaches, barriers, and enabling factors for each intervention were also analyzed to provide a holistic understanding. By triangulating evidence from diverse sources, this review aimed to present a well-rounded narrative of how behavioral strategies contribute to the broader malaria control agenda.

## 4. Ethical approval

Not applicable as this is a narrative paper.

## 5. Rationale

Malaria remains a significant public health challenge, particularly in sub-Saharan Africa and other tropical regions. Despite advancements in medical treatments and preventive measures, the persistence of malaria highlights the need for comprehensive control strategies. Behavioral health interventions, which include community education, behavior change communication, and the promotion of preventive practices (e.g., use of ITNs and IRS), are critical components of malaria control programs. These interventions aim to modify individual and community behaviors to reduce malaria transmission and improve health outcomes. Understanding the efficacy of these interventions is crucial for policymakers, health practitioners, and researchers to design effective malaria control programs. Additionally, examining the implementation processes can reveal insights into the facilitators and barriers to success, helping to optimize the deployment of these interventions in diverse settings. This review will contribute to the body of knowledge by systematically analyzing the impact of behavioral health interventions on malaria control and identifying strategies that have been most effective in real-world settings.

## 6. Linking malaria pathogenesis to behavioral prevention strategies

Malaria pathogenesis provides a crucial foundation for understanding and optimizing prevention strategies, especially when integrating behavioral interventions. The disease is caused by *Plasmodium* parasites, primarily *Plasmodium falciparum*, which are transmitted to humans through the bite of infected *Anopheles* mosquitoes. Once in the bloodstream, the parasite undergoes a complex life cycle involving the liver and red blood cells. This process includes an asymptomatic liver stage and a symptomatic blood stage, which together contribute to the clinical manifestations of malaria, ranging from fever and malaise to severe complications such as cerebral malaria and death. Each stage of the parasite’s life cycle offers an opportunity for targeted intervention, and behavioral strategies are critical in enhancing the effectiveness of biomedical tools at these points. For instance, during the initial phase when sporozoites are introduced through mosquito bites, personal and community behaviors – such as the consistent use of ITNs and participation in IRS campaigns – serve as primary barriers to infection. However, evidence from the WHO suggests that access does not always equate to usage. Cultural beliefs, misconceptions, discomfort due to heat, and a lack of perceived risk all influence ITN utilization, highlighting the need for behaviorally tailored health education campaigns.^[13]^ As the parasite transitions to the blood stage, rapid and appropriate treatment becomes essential to interrupt the cycle of transmission and reduce morbidity. Here, behavioral health interventions play a pivotal role in encouraging timely care-seeking, discouraging self-medication, and improving adherence to artemisinin-based combination therapies (ACTs). Without adequate awareness and support, delays in treatment can lead to severe outcomes and continued community transmission.^[1,2]^ Moreover, asymptomatic carriers – individuals who harbor the parasite without clinical symptoms – pose a silent challenge to malaria control efforts. These carriers often fail to seek treatment, thereby sustaining the reservoir of infection within communities. Behavior change communication (BCC) campaigns aimed at increasing community-level knowledge and promoting proactive health behavior can bridge this gap, enhancing surveillance and prompting early diagnosis.^[3–5]^ In essence, a clear understanding of malaria pathogenesis underscores the importance of synchronizing prevention efforts with human behavior. Biomedical interventions, while effective, must be supported by community-driven behavioral changes to ensure consistent application, proper use, and long-term impact. Embedding behavioral science within malaria control frameworks ensures that prevention strategies are not only scientifically sound but also socially and culturally responsive, thereby accelerating progress toward global elimination goals.^[6–8]^

## 7. Behavioral interventions and biomedical linkages

Behavioral strategies are not merely adjuncts to biomedical interventions in malaria control – they are central to interrupting the disease cycle, mitigating pathogenesis, and improving clinical outcomes. The success of antimalarial therapies, preventive tools, and vector control measures is profoundly influenced by individual and community behaviors. This section explores the mechanisms through which key behavioral interventions – such as prompt treatment-seeking, adherence to chemoprophylaxis, and correct use of ITNs – contribute to altering the course of malaria infection and disease severity.^[9]^

### 7.1. Prompt treatment-seeking and disease progression

Early recognition of malaria symptoms and immediate health-seeking behavior are critical to preventing the progression from uncomplicated malaria to severe and potentially fatal outcomes, particularly in pregnant women and children. Delayed treatment increases parasite biomass, exacerbates inflammation, and elevates the risk of complications such as cerebral malaria, anemia, and multi-organ failure. Behavioral interventions that improve awareness of early symptoms (e.g., fever, chills, vomiting) and the urgency of care-seeking – especially in remote and rural communities – can lead to earlier diagnosis and timely administration of ACTs, which are most effective during the early stages of infection.^[10,11]^

### 7.2. Adherence to prophylaxis and immune modulation

In endemic regions, adherence to chemoprophylaxis (e.g., sulfadoxine-pyrimethamine for intermittent preventive treatment in pregnancy or seasonal malaria chemoprevention in children) plays a significant role in reducing parasite burden and modulating host immune response. Inconsistent or incomplete adherence diminishes the prophylactic effect, allowing for subclinical infections that may trigger chronic immune activation or recrudescence. Behavioral interventions that target health education, address myths about side effects, and reinforce dosing schedules through reminders or peer support have been shown to improve compliance and maintain protective drug levels, thereby lowering the risk of severe outcomes.^[12,13]^

### 7.3. Use of vector control tools and infection prevention

Behavioral practices surrounding the use of ITNs and IRS directly influence exposure to infectious mosquito bites. Despite wide distribution, net ownership does not always translate into proper or consistent use due to factors such as discomfort, heat, or perceived lack of necessity. Effective behavioral strategies – including community demonstrations, school-based education, and culturally tailored messaging – can increase nightly use, reduce mosquito-human contact, and ultimately decrease parasitemia rates in vulnerable populations.^[14,15]^

### 7.4. Health-seeking behavior during pregnancy and pediatric vulnerability

Pregnant women and children under 5 are at heightened risk of severe malaria due to altered immunity and physiological vulnerability. Behavioral interventions that promote routine antenatal care (ANC) attendance ensure access to preventive tools such as ITNs, intermittent preventive treatment in pregnancy, and diagnostic screening. Health literacy programs that encourage early ANC booking and continuity of care directly enhance maternal and neonatal outcomes by reducing placental parasitemia, low birth weight, and preterm labor. Similarly, child-focused interventions, such as caregiver education and community-based case management, can ensure rapid response to febrile illness and reduce mortality.^[16,17]^

## 8. The role of behavior in malaria transmission and control

Behavior plays a pivotal yet often underappreciated role in both the transmission and control of malaria. While biomedical interventions such as ITNs, IRS, ACTs, and intermittent preventive treatments have significantly reduced global malaria burdens, their effectiveness is highly dependent on human behavior. The consistent and correct use of these interventions, adherence to treatment regimens, and timely health-seeking actions are all shaped by individual choices, cultural beliefs, community norms, and socio-economic factors.^[14–16]^ For instance, despite increased availability of ITNs across endemic regions, their usage remains inconsistent. Factors such as discomfort during hot seasons, perceived invulnerability to malaria, lack of awareness, and myths surrounding the use of nets contribute to underutilization. According to WHO estimates, although approximately 68% of populations at risk in sub-Saharan Africa have access to ITNs, actual usage often falls short of this figure. Similarly, IRS campaigns frequently face resistance due to concerns over chemical exposure, damage to household items, or lack of perceived benefit, reducing the reach and impact of these interventions.^[17–19]^

Furthermore, delayed or inappropriate care-seeking behavior continues to hinder early diagnosis and treatment, allowing the malaria parasite to replicate unchecked within human hosts. In some cases, individuals resort to traditional medicine or self-medication rather than visiting health facilities, a behavior often driven by poverty, geographic inaccessibility, or mistrust in the healthcare system. These delays can contribute to severe disease progression, increased mortality, and sustained community-level transmission.^[20]^ Behavior also influences the success of mass drug administration programs and community engagement initiatives. Community trust, perceptions of drug safety, and understanding of malaria’s transmission cycle all affect the willingness to participate in preventive strategies. Education campaigns that are culturally appropriate and linguistically accessible have been shown to significantly improve compliance and long-term adoption of protective behaviors.^[21]^ Ultimately, malaria is not merely a biological challenge but a social one. Control efforts must extend beyond distributing commodities to understanding and addressing the human behaviors that determine their success. Integrating behavioral science into malaria programs – through health education, BCC, and community empowerment – offers a complementary and necessary pathway toward malaria elimination. Recognizing the centrality of human behavior provides a holistic lens through which to enhance the design, implementation, and sustainability of malaria control strategies worldwide.^[22,23]^

## 9. The Role of behavioral health interventions in malaria control

### 9.1. Community education

Community education is a cornerstone of behavioral health interventions in malaria control.^[13]^ It involves disseminating information and raising awareness about malaria transmission, prevention, and treatment among at-risk populations. This approach leverages various channels, including schools, community centers, health clinics, and media platforms, to reach diverse audiences. Effective community education programs are tailored to the specific cultural and social contexts of the communities they serve, ensuring that the information is accessible, relevant, and actionable.

### 9.2. Objectives of community education

The primary objectives of community education in malaria control are to:

1. **Increase awareness**: Educate communities about how malaria is transmitted, the symptoms of the disease, and the importance of early diagnosis and treatment.
2. **Promote preventive measures**: Encourage the consistent use of ITNs, IRS, and other preventive measures.
3. **Improve treatment-seeking behavior**: Motivate individuals to seek prompt and appropriate medical treatment when malaria symptoms appear.
4. **Foster community involvement**: Engage community members in malaria control activities to build local capacity and sustain efforts.

### 9.3. Methods of community education

1. **School-based programs**: Schools provide an effective platform for educating children and, by extension, their families. Programs may include classroom lessons, interactive activities, and distribution of educational materials.
2. **Community health talks**: Health talks conducted at community centers, marketplaces, and religious gatherings can reach large audiences. These sessions often involve local health workers or volunteers who provide information and answer questions about malaria.
3. **Media campaigns**: Mass media campaigns utilize radio, television, newspapers, and social media to disseminate information widely. These campaigns can reach a broad audience and reinforce key messages through repetition.
4. **Printed materials**: Pamphlets, posters, and brochures distributed in public places, health facilities, and homes can serve as constant reminders of malaria prevention practices.
5. **Interactive workshops**: Workshops and training sessions for community leaders, health workers, and volunteers can help to cascade knowledge throughout the community. These sessions often include practical demonstrations on the use of ITNs and other preventive measures.

### 9.4. Efficacy of community education

Community education has been shown to significantly improve knowledge and behaviors related to malaria prevention and treatment.^[14]^ For instance, a study in rural Kenya found that educational campaigns increased ITN usage from 30% to 70% over 3 years.^[15]^ Another example is from Ethiopia, where a comprehensive community education program led to increased awareness and improved treatment-seeking behavior, resulting in reduced malaria incidence.^[16]^

The success of community education programs depends on several factors:

- **Cultural relevance**: Tailoring messages to align with local beliefs, practices, and languages increases acceptance and impact.
- **Community engagement**: Involving local leaders and community members in planning and delivering education activities fosters ownership and trust.
- **Sustainability**: Ensuring that education efforts are continuous and supported by local resources and capacity-building initiatives.

### 9.5. Challenges in implementation

1. **Cultural barriers**: Misconceptions about malaria transmission and treatment can hinder the effectiveness of education efforts. Some communities may have traditional beliefs that conflict with modern medical advice.
2. **Resource constraints**: Limited funding, personnel, and materials can restrict the reach and frequency of educational activities.
3. **Access issues**: In remote or conflict-affected areas, reaching target populations with educational messages can be challenging.

### 9.6. Strategies for overcoming challenges

1. **Community involvement**: Engaging community leaders and members in designing and delivering education programs ensures cultural appropriateness and increases buy-in.
2. **Partnerships and collaboration**: Collaborating with nongovernmental organizations (NGOs), educational institutions, and government agencies can enhance resources and expertise available for community education.
3. **Innovative approaches**: Utilizing mobile health (mHealth) applications, social media, and other digital platforms can extend the reach of education efforts, especially in hard-to-reach areas.

### 9.7. Case studies

1. **Kenya**: A community education program that involved local health workers conducting home visits and distributing educational materials led to a substantial increase in ITN usage and early treatment-seeking behavior.^[17]^
2. **Ethiopia**: In Ethiopia, a combined approach using school-based programs, community health talks, and media campaigns resulted in increased awareness and improved malaria prevention practices.^[18]^

## 10. Behavioral change communication

BCC is a strategic approach used in public health to promote positive behaviors and mitigate harmful practices that contribute to disease transmission.^[19]^ In the context of malaria control, BCC plays a critical role in encouraging behaviors that prevent malaria transmission and improve treatment outcomes. By leveraging various communication methods, BCC aims to influence individual and community behaviors, fostering a supportive environment for malaria prevention and control.

### 10.1. Objectives of BCC in malaria control

The primary objectives of BCC in malaria control are to:

1. **Increase knowledge**: Enhance understanding of malaria transmission, symptoms, prevention, and treatment among target populations.
2. **Promote preventive behaviors**: Encourage the consistent use of ITNs, IRS, and other preventive measures.
3. **Improve treatment-seeking behavior**: Motivate individuals to seek prompt and appropriate medical care at the first signs of malaria symptoms.
4. **Reduce risky practices**: Discourage behaviors that increase the risk of malaria, such as improper use of ITNs and delayed treatment-seeking.

### 10.2. Methods of BCC

1. **Mass media campaigns**: Utilizing radio, television, newspapers, and social media to disseminate information widely. These campaigns can reach broad audiences and reinforce key messages through repeated exposure.
2. **Interpersonal communication**: Engaging individuals and small groups in direct conversations about malaria prevention and treatment. This method includes counseling sessions, peer education, and community dialogs.
3. **Community mobilization**: Involving community members and leaders in malaria control activities to foster collective action and ownership. This can include organizing community meetings, health fairs, and participatory workshops.
4. **Theatrical performances and storytelling**: Using local theater groups, storytelling, and role-playing to convey messages in a culturally relevant and engaging manner. These methods can effectively capture attention and promote behavior change.
5. **Print and digital materials**: Distributing brochures, posters, and leaflets in public places, health facilities, and homes. Digital platforms, including mobile health (mHealth) applications and social media, can also be used to share information and engage with the community.

### 10.3. Efficacy of BCC

BCC has been shown to be effective in changing behaviors and reducing malaria transmission.^[20]^ Successful BCC interventions are characterized by their ability to resonate with the target audience and address specific behavioral determinants. For example, the “Zinduka! Malaria Haikubaliki” campaign in Tanzania effectively used radio and television broadcasts, posters, and community meetings to promote ITN use and timely treatment-seeking, resulting in increased ITN usage and a decrease in malaria incidence. Identifying and tailoring messages to specific segments of the population based on factors such as age, gender, socio-economic status, and cultural beliefs.^[21]^ Ensuring that messages and delivery methods are culturally appropriate and resonate with the target audience. This includes using local languages, idioms, and culturally significant symbols. Repeated exposure to key messages through multiple channels reinforces learning and encourages behavior change. Creating opportunities for dialogue and feedback to address concerns, misconceptions, and barriers to behavior change. Continuously assessing the impact of BCC activities to refine strategies and improve effectiveness. This involves collecting data on behavior changes and health outcomes, and adjusting campaigns based on findings.

Deep-rooted beliefs and practices can be resistant to change. Misconceptions about malaria transmission and treatment can undermine BCC efforts.^[22]^ Insufficient funding, trained personnel, and materials can limit the scope and reach of BCC campaigns. Reaching remote or conflict-affected areas with BCC messages can be challenging. Overexposure to health messages can lead to desensitization and reduced impact. Engaging community leaders and members in the design and delivery of BCC activities ensures cultural relevance and increases acceptance.^[23]^ Collaborating with nongovernmental organizations (NGOs), government agencies, and local institutions can enhance resources and expertise. Utilizing digital platforms, social media, and mobile health (mHealth) applications can extend the reach of BCC efforts and engage hard-to-reach populations. Investing in training for health workers and community volunteers to enhance their communication skills and ability to deliver effective BCC messages.

### 10.4. Case studies

1. **Tanzania**: The “Zinduka! Malaria Haikubaliki” campaign successfully increased ITN usage and reduced malaria incidence by using a combination of mass media, community meetings, and print materials to promote malaria prevention practices.^[24]^
2. **Uganda**: A BCC initiative that trained CHWs to conduct home visits and provide education on ITN use and prompt treatment-seeking behavior resulted in significant improvements in malaria prevention practices and reduced malaria cases in targeted communities.^[25,26]^

## 11. Community-based interventions

Community-based interventions play a pivotal role in malaria control efforts by engaging local communities in the planning, implementation, and evaluation of malaria prevention and treatment strategies.^[27,28]^ These interventions recognize the importance of leveraging local knowledge, resources, and social networks to address the complex challenges of malaria transmission and control. By fostering community ownership and participation, community-based interventions can enhance the effectiveness, sustainability, and scalability of malaria control programs.^[29,30]^

Involving community members in all stages of program design, implementation, and evaluation to ensure relevance, acceptance, and sustainability. Providing training and support to CHWs, volunteers, and community leaders to enhance their knowledge, skills, and confidence in delivering malaria control interventions.^[31,32]^ Collaborating with local stakeholders, including government agencies, nongovernmental organizations (NGOs), and community-based organizations, to leverage resources, expertise, and networks.^[33]^ Adapting interventions to the specific needs, preferences, and cultural contexts of target communities to maximize effectiveness and minimize resistance. Establishing robust monitoring and evaluation (M&E) systems to track progress, identify challenges, and inform programmatic adjustments.^[34]^

### 11.1. Objectives of community-based interventions

The primary objectives of community-based interventions in malaria control are to:

1. **Empower communities**: Build local capacity and leadership to take ownership of malaria control activities.
2. **Promote participation**: Engage community members in identifying needs, setting priorities, and implementing solutions.
3. **Improve access to services**: Increase access to malaria prevention, diagnosis, and treatment services at the community level.
4. **Strengthen social support**: Foster supportive environments that encourage adherence to preventive measures and treatment protocols.

### 11.2. Methods of community-based interventions

1. **CHWs**: Train and deploy community members as CHWs to deliver education, distribute ITNs, provide antimalarial treatments, and conduct rapid diagnostic tests in their communities.^[35]^
2. **Volunteer networks**: Mobilize volunteers from within communities to support malaria control activities, including advocacy, education, and community mobilization efforts.^[36]^
3. **Community health committees**: Establish committees comprising community members, health workers, and local leaders to oversee and coordinate malaria control activities at the community level.^[37]^
4. **Home visits**: Conduct home visits by CHWs or volunteers to provide personalized education, distribute ITNs, and reinforce malaria prevention messages.^[38]^
5. **Community dialogue and advocacy**: Organize community meetings, focus group discussions, and advocacy campaigns to raise awareness, address misconceptions, and mobilize support for malaria control efforts.^[39]^

### 11.3. Efficacy of community-based interventions

Community-based interventions have demonstrated effectiveness in reducing malaria transmission and morbidity in diverse settings. For example, a study in Zambia found that training and deploying CHWs to provide malaria prevention and treatment services at the community level led to a significant reduction in malaria-related mortality.^[40]^ Similarly, in Ghana, community-based distribution of ITNs through volunteer networks resulted in increased ITN usage and decreased malaria incidence.^[41]^

## 12. Implementation barriers and adaptive strategies

Effective behavioral health interventions in malaria control often face a range of implementation challenges, particularly in resource-limited and culturally diverse settings. These barriers can undermine even the most evidence-based strategies if not adequately addressed. This section outlines key obstacles to implementation and highlights adaptive strategies that have shown promise in overcoming them.^[42,43]^

### 12.1. Cultural beliefs and misinformation

In many malaria-endemic regions, deeply rooted cultural beliefs and misconceptions about the disease’s etiology – such as spiritual causes, witchcraft, or environmental myths – hinder the uptake of biomedical and behavioral interventions. Resistance to IRS and distrust of ITNs may stem from fears of infertility, toxicity, or political suspicion. Culturally sensitive engagement is critical. Involving respected local leaders, traditional healers, and religious figures in health education efforts enhances trust and message legitimacy. Tailoring messages in local languages and using storytelling, drama, or oral traditions can improve message retention and acceptance.^[44,45]^

### 12.2. Limited health literacy and communication gaps

Low levels of literacy and health knowledge can result in misinterpretation of health messages or incorrect use of malaria prevention tools. Printed materials may be ineffective in populations with limited reading proficiency. Using visual, audio, and interactive communication tools – such as radio programs, pictorial leaflets, mobile loudspeaker announcements, and community theater – can bridge comprehension gaps. CHWs trained in participatory communication techniques can foster dialogue and clarify misconceptions.^[46,47]^

### 12.3. Socio-economic constraints

Poverty often limits access to preventive tools and health services. Even when ITNs are distributed for free, the indirect costs of care (e.g., transportation, time off work, or clinic fees) discourage treatment-seeking behavior. Behavioral interventions must be paired with social support mechanisms. Conditional cash transfers, transportation vouchers, and community-based health insurance schemes can help remove financial barriers. Moreover, aligning malaria interventions with broader poverty reduction efforts increases sustainability and equity.^[48–50]^

### 12.4. Weak health system infrastructure

Insufficient staffing, supply chain failures, and poorly trained health personnel can compromise the delivery and effectiveness of behavior-focused malaria programs. Capacity-building initiatives, such as training CHWs in BCC, can decentralize service delivery and increase community reach. Digital tools like mobile reporting systems or SMS-based health tips can enhance efficiency and monitoring.^[51,52]^

### 12.5. Inconsistent program funding and political support

Donor-dependent programs often suffer from discontinuity when funding cycles end. A lack of political commitment can further undermine behavioral health investments. Advocating for domestic resource mobilization and integrating behavioral health into national malaria control strategies can enhance program sustainability. Multi-sectoral collaboration – including education, housing, and agriculture – ensures broader buy-in and resource pooling.^[43,44]^

### 12.6. Monitoring and evaluation challenges

Behavioral outcomes are inherently complex and often slow to manifest. Poorly designed evaluation frameworks may fail to capture nuanced changes in attitudes, beliefs, or long-term behaviors.^[53,54]^ Mixed-methods approaches that combine quantitative indicators (e.g., ITN usage rates) with qualitative tools (e.g., focus group discussions, community feedback) offer a more comprehensive evaluation of behavioral interventions. Community-led monitoring models can also enhance accountability and local ownership.^[55–57]^

## 13. Conclusion

Malaria control efforts rely on a multifaceted approach that encompasses technological advancements, behavioral health interventions, and community-based strategies. Technological advancements in malaria control, including novel vector control methods and climate change adaptation strategies, offer promising avenues for reducing malaria transmission. Innovations such as genetically modified mosquitoes, spatial repellents, and digital surveillance tools hold the potential to complement traditional control measures and overcome emerging challenges posed by vector resistance and environmental changes. Behavioral health interventions play a crucial role in shaping individual and community behaviors related to malaria prevention, diagnosis, and treatment. Community education, BCC, and community-based interventions have demonstrated effectiveness in improving malaria knowledge, promoting preventive behaviors, and enhancing treatment-seeking practices. By addressing cultural barriers, promoting community engagement, and tailoring interventions to local contexts, behavioral health approaches contribute to the success and sustainability of malaria control efforts. Community-based interventions harness the power of local communities in driving malaria control activities. Through the empowerment of CHWs, volunteer networks, and community health committees, these interventions promote community ownership, strengthen health systems, and improve access to malaria prevention and treatment services. By leveraging local knowledge, resources, and social networks, community-based interventions enhance the reach, effectiveness, and sustainability of malaria control programs.

## Author contributions

**Conceptualization:** Emmanuel Ifeanyi Obeagu.

**Methodology:** Emmanuel Ifeanyi Obeagu, Getrude Uzoma Obeagu.

**Resources:** Emmanuel Ifeanyi Obeagu.

**Supervision:** Emmanuel Ifeanyi Obeagu.

**Validation:** Emmanuel Ifeanyi Obeagu, Getrude Uzoma Obeagu, Michael Unata Iduh.

**Visualization:** Emmanuel Ifeanyi Obeagu, Getrude Uzoma Obeagu, Michael Unata Iduh.

**Writing** – **original draft:** Emmanuel Ifeanyi Obeagu, Getrude Uzoma Obeagu, Michael Unata Iduh.

**Writing** – **review & editing:** Emmanuel Ifeanyi Obeagu, Getrude Uzoma Obeagu, Michael Unata Iduh.
